# Mpox Vaccination in Non-Endemic Countries: Considerations for Public Health and Policy

**DOI:** 10.3390/vaccines11091406

**Published:** 2023-08-23

**Authors:** Pietro Ferrara

**Affiliations:** 1 Center for Public Health Research, University of Milan–Bicocca, 20900 Monza, Italy; pietro.ferrara@unimib.it; 2 IRCCS Istituto Auxologico Italiano, 20145 Milano, Italy

## 1. Foreword

The disease mpox (formerly monkeypox) is a zoonotic viral disease caused by a virus belonging to the *Orthopoxvirus*, the same genus as smallpox. It is primarily found in tropical rainforest areas of central and west Africa [1,2]. Although mpox cases have historically been limited to these regions, recent outbreaks in non-endemic countries have raised concerns about the need for appropriate public health policies. In 2022–2023, cases have spread worldwide via direct human-to-human transmission, primarily through close contact, linked to the clade IIb of the mpox virus [1]. Two other distinct lineages have been identified: clade I is found in the Congo Basin, transmitted by rodents, and can result in human mortality of up to 10%; clade IIa is prevalent in west Africa, characterized by lower mortality rates, and is likewise a zoonotic disease [3]. As the number of cases increased in countries not typically affected by the virus, the World Health Organization (WHO) International Health Regulations (2005) (IHR) Emergency Committee officially designated the worldwide outbreak of mpox as a global health emergency on 23 July 2022, which officially terminated in May 2023 [1]. As of August 2023, more than 89,000 confirmed cases have been documented across more than 113 countries [4].

To tackle this emergency, vaccines and treatments developed for smallpox, sanctioned for utilization in specific nations, may found application in addressing mpox. While antiviral agents displaying effectiveness against poxviruses (such as brincidofovir, cidofovir, and tecovirimat) are primarily reserved for treating severe mpox cases or immunocompromised patients, to address the global outbreak, specific preventive measures principally include the isolation of cases and administration of smallpox/mpox vaccines—i.e., ACAM2000^®^ orthopoxvirus and JYNNEOS™ (also known as Imvamune or Imvanex) vaccines—targeting at-risk populations [1,5,6,7,8]. These include health workers at risk of exposure, men who have sex with men (MSM), people with multiple sex partners, and sex workers [1]. Furthermore, to reduce the risk and severity of mpox infection, people may be vaccinated after exposure to mpox virus, as post-exposure prophylaxis, within 4 days of contact with an infected person (or within up to 14 days if there are no symptoms) [1,8].

In this frame, the presented Special Issues of *Vaccines* offers interesting insights into fundamental research on mpox vaccination from a public health perspective [9]. An important collection of contributions and studies has been presented. Each of them contributes an important piece to the evolving challenges in vaccination strategies, and suggests future topics for further research in the field.

## 2. The Public Health Considerations in Mpox Vaccination

Overall, four original articles were submitted to this Special Issue to be considered for publication, and all were accepted for publication after evaluation by the Guest Editor and peer-review experts. The first Special Issue paper, by Zucman et al., highlights mpox vaccine hesitancy in a sample of 155 French MSM living with human immunodeficiency virus (HIV) infection or taking HIV pre-exposure prophylaxis (PrEP), who have an increased risk of infection and increased disease consequences [10]. Vaccine hesitancy involves a cautious or uncertain attitude toward accepting vaccines, particularly a ‘new’ vaccine such as mpox. This hesitancy can be fueled by low levels of the perceived severity of the disease, a low level of institutional trust, and concerns about the development process and potential side effects [11]. Zucman et al. found that one-third of participants declared their hesitancy to be vaccinated against mpox. PrEP users who expressed worries about receiving an excessive number of vaccines (considering the concomitant COVID-19 vaccination campaigns [12]) showed a lower vaccine acceptance, while a greater number of sexual partners was significantly associated with higher acceptance in both PrEP and HIV groups. Overall, the results from this study remarked that addressing mpox vaccine hesitancy requires transparent communication, as well as sharing accurate information about the vaccine’s benefits and safety profile, and its role in safeguarding public health [10].

Successful prevention programs, including vaccination, strictly depend on public knowledge and perceptions of the disease and its transmission. In this regard, Temsah et al. conducted research investigating these aspects in the general public in Saudi Arabia (n = 1546), and healthcare workers (HCWs; n = 1130), during the first month of the WHO alert. Overall, 61.3% of the public and 74.2% of HCWs showed interest in seeking more information about mpox, while current knowledge was higher in those concerned by the mpox risk. Regarding vaccination, HCWs showed poor knowledge regarding disease presentation and vaccination [13]. Similarly, in July 2022, Ahmed et al. investigated the knowledge, perspective, and apprehension of mpox in the Kurdish population in Iraq, who exhibited a moderate-to-low level of awareness, a neutral stance, and a moderate-to-low level of concern regarding the disease [14]. Both studies pinpointed areas for enhancements in the awareness and knowledge gaps regarding mpox among both the general public and HCWs, with a particular focus on the latter. Such information should be harnessed by policymakers in awareness campaigns aimed at curtailing disease transmission and promoting vaccination. The studies underscore the necessity for continued research into similar emerging infectious disease threats.

Appropriate mpox prevention and control strategies are also based on accurate knowledge of its geospatial distribution through mathematical modelling, which allows for a description the epidemic dynamics, in addition to investigations of the consistency and effectiveness of measures, and prediction of possible scenarios. In particular, compartmental models are key epidemiological tools applied to the study of the transmission of infectious diseases, in which people are assigned to single compartments—such as Susceptible, Infectious, or Recovered (S, I, or R)—and may move across compartments [15]. In their research, Liao et al. [16] estimated the basic/effective reproductive number (R_0_/R_e_) and assessed the epidemic spread of monkeypox across the globe though a susceptible–infected–recovered (SIR) model with a directed acyclic graph, using data retrieved up to 23 September 2022. According to this study, the maximum estimated R_0_/R_e_ was 1.16 (95% confidence interval [CI], 1.15–1.17) in the United States, 1.20 (95% CI, 1.20–1.20) in Spain, 1.34 (95% CI, 1.34–1.35) in Brazil, 1.33 (95% CI, 1.33–1.33) in the United Kingdom, and 2.52 (95% CI, 2.41–2.66) in the Democratic Republic of the Congo, respectively. The values of R_0_/R_e_ were below 1 after August 2022. In sum, the authors highlighted the successful control of the global mpox outbreak, starting from the first epidemic months, by virtue of public health measures and the existence of effective vaccines. Finally, Liao et al. [16] remarked that ongoing surveillance of the outbreak remains imperative as time progresses. 

## 3. Conclusions

While mpox was known to be primarily confined to Central and West Africa, in 2022, a significant global outbreak was recognized in numerous countries where the disease is not typically prevalent. The occurrence of outbreaks and sporadic cases necessitates thoughtful public health strategies. Targeted vaccination, robust surveillance, and public awareness efforts can help mitigate the risks associated with mpox, ensuring prompt detection, containment, and the prevention of further spread. Collaboration among nations and continuous research is essential for strengthening our understanding and control of this disease in endemic and non-endemic areas.
